# Development and validation of clinical profiles of patients hospitalized due to behavioral and psychological symptoms of dementia

**DOI:** 10.1186/s12888-016-0966-7

**Published:** 2016-07-22

**Authors:** Claudia Ortoleva Bucher, Nicole Dubuc, Armin von Gunten, Lise Trottier, Diane Morin

**Affiliations:** Institute of Higher Education and Research in Healthcare (IUFRS), Faculty of Biology and Medicine, University of Lausanne and Lausanne University Hospital, Route de la Corniche 10, 1010 Lausanne, Switzerland; Research Centre on Aging, Sherbrooke University Geriatrics Institute, Quebec, Canada; School of Nursing, Faculty of Medicine and Health Sciences, Université de Sherbrooke, Quebec, Canada; Service of Old Age Psychiatry, Department of Psychiatry, Lausanne University Hospital, Lausanne, Switzerland; Faculty of Nursing Sciences, Laval University, Quebec, Canada

**Keywords:** Aged, Dementia, Psychogeriatrics, Inpatients, Clustering

## Abstract

**Background:**

Patients hospitalized on acute psychogeriatric wards are a heterogeneous population. Cluster analysis is a useful statistical method for partitioning a sample of patients into well separated groups of patients who present common characteristics. Several patient profile studies exist, but they are not adapted to acutely hospitalized psychogeriatric patients with cognitive impairment. The present study aims to partition patients hospitalized due to behavioral and psychological symptoms of dementia into profiles based on a global evaluation of mental health using cluster analysis.

**Methods:**

Using nine of the 13 items from the Health of the Nation Outcome Scales for elderly people (HoNOS65+), data were collected from a sample of 542 inpatients with dementia who were hospitalized between 2011 and 2014 in acute psychogeriatric wards of a Swiss university hospital. An optimal clustering solution was generated to represent various profiles, by using a mixed approach combining hierarchical and non-hierarchical (k-means) cluster analyses associated with a split-sample cross-validation. The quality of the clustering solution was evaluated based on a cross-validation, on a k-means method with 100 random initial seeds, on validation indexes, and on clinical interpretation.

**Results:**

The final solution consisted of four clinically distinct and homogeneous profiles labeled (1) *BPSD-affective*, (2) *BPSD-functional*, (3) *BPSD-somatic* and (4) *BPSD-psychotic* according to their predominant clinical features. The four profiles differed in cognitive status, length of hospital stay, and legal admission status.

**Conclusion:**

In the present study, clustering methods allowed us to identify four profiles with distinctive characteristics. This clustering solution may be developed into a classification system that may allow clinicians to differentiate patient needs in order to promptly identify tailored interventions and promote better allocation of available resources.

**Electronic supplementary material:**

The online version of this article (doi:10.1186/s12888-016-0966-7) contains supplementary material, which is available to authorized users.

## Background

Around 110,000 people currently suffer from dementia in Switzerland [1]. By 2050, demographic trends suggest that its prevalence will triple [2]. The overall annual direct and indirect costs of dementia in Switzerland amount to CHF 6.9 billion [1]. Thus, dementia already has a significant impact on healthcare systems; its complications include individual suffering, family burdens, increasing needs for nursing and interprofessional care, and longer hospital stays [3–5]. Behavioral and psychological symptoms of dementia (BPSD) are universal and affect nearly all patients over the course of dementia [6–8]. They have negative consequences such as high level of caregiver distress [9, 10], poor quality of life not only for patients but also for their formal and informal caregivers [11–13], and a significant increase in costs [14, 15]. Moreover, BPSD constitute a major cause of hospital admissions [16].

It is commonly considered that psychogeriatric inpatients with dementia constitute a heterogeneous population and some research has already focused on BPSD profiles in dementia [17–20]. However, to date, the different patient profile studies combining somatic and psychiatric problems with socio-relational information, clinical evolution and care management are poorly described. Consequently, better knowledge regarding patients’ profiles in this type of setting is needed to better inform future health policy decisions regarding dementia care [21].

To better target the needs of these patients and, thus, more effectively adjust the interventions and resources required, a multidimensional assessment of health is essential; it should include the dimensions of psychopathology, social functioning, cognition, pain/discomfort and other physical features as well as satisfaction [22]. Although using the overall score from a multidimensional assessment tool can give an indication as to the severity of a clinical situation, it does not provide a true representation of the various components involved. Indeed, a similar score on a given scale can be the result of different combinations of simultaneous health conditions. Cluster analysis is a useful way of identifying the different profiles present in a heterogeneous population. Identifying groups of people who share similar characteristics will help to better understand those characteristics [23, 24]. Attributing a label to each group allows professionals to use a common language and facilitates discussion and information sharing. Also, in the healthcare sector, classifications developed by cluster analyses gather individuals who require the same resources and, until now, they were mainly created in order to facilitate the efficient attribution of limited resources [25, 26]. Knowledge about people’s affiliation to a specific profile offers refined information that helps to determine the most efficient interventions required in various situations and also enables the development of a classification system. For example, the Iso-SMAF profiles, developed in Canada, is a disability-based classification system for the management of long-term care needs in an integrated service delivery system [27].

So far, several studies have developed classification systems for psychiatry based on the prediction of a dependant variable using statistical methods such as regression tree analysis, namely the Psychiatric Patient Classification System [28], the Patient Casemix Classification [29], the Australian National Subacute and Non-Acute Patient classification (AN-SNAP, Versions I and II) [30, 31], the Long-stay Psychiatric Patient Classification system [32], the System for Classification of In-Patient Psychiatry (SCIPP) [33], the Mental Health Care Clusters [34] and the mental health Classification And Outcomes Study (NZ CAOS) [35, 36]. The characteristics of these seven classification systems have been summarized in Additional file 1. Considering that the development of a new classification system is an arduous task, the use of an existing and operational foreign system could have been judicious. However, the above classifications were developed to correspond to the specific healthcare system in which they were developed. When an attempt was made to transfer the American Resource Utilization Groups specially designed to estimate the costs of long term institutions to England, it was unsuccessful [37]. Indeed, American, but not British long-term care, is provided in rehabilitation settings, illustrating the importance of the fit between healthcare systems to consider the transfer of a foreign classification system. In the context of this study, the Canton of Vaud has a specific healthcare organization and has developed acute psychogeriatric wards specifically dedicated to the management of patients with BPSD; these wards are located in three different regions in an effort to offer proximity services to patients and their families. Each of the classifications reviewed was developed for specific psychiatric organizations and did not accurately match the specificities of psychogeriatric inpatients in the Swiss French context. Furthermore, several points in the classification development have to be taken into consideration concerning the statistical methods, the variables to be included in the analysis and the sample*.*

Firstly, all the classifications, except the Mental Health Care Clusters [34] and the Psychiatric Patient Classification System [28], use costs as a dependent variable in their development; however, from a clinical point of view, this should not be the case [38]. Indeed, when groups are defined by the amount of resources they require, classification becomes more difficult especially when a new intervention which requires a change in resources is used on a specific group. By defining groups based on clinical features and clinical relevance, the introduction of new procedures will be guided by the characteristics of the group.

Secondly, using diagnosis as a classification variable - as in the Psychiatric Patient Classification System [28], the Patient Casemix Classification [29], and the AN-SNAP [30] - is not adequate for aged psychiatric inpatients. Indeed, it is difficult to classify these patients when the mental health diagnosis is not the main reason for hospitalization. It is common for psychogeriatric patients to be hospitalized when home care, nursing home care or care in a general hospital become too difficult. This can be the case with a patient with behavioral disorders, a caregiver suffering from exhaustion, or the failure/refusal of care.

Thirdly, none of the classifications reviewed was developed to meet the specific needs of older people with cognitive impairment hospitalized in geriatric psychiatry units. All of them were developed on the basis of samples composed of adults and aged patients and the NZ CAOS [35, 36] was developed for all ages. The selection of the sample for the development of classification is essential and must be carefully planned [23, 24]. If individuals of different ages constitute the sample, dissimilarities across the different age groups will cancel out differences among individuals of the same age. Thus, psychogeriatric inpatients with dementia should be the only subjects to be included in the classification developement procedure.

For all the aforementioned reasons, none of the existing and operational foreign classification systems could be applied to our specific population of aged patients with cognitive impairment hospitalized in acute geriatric psychiatry units. Moreover, there is a need to develop a partition of these patients using clustering methods rather than classification methods based on the prediction of costs. Of all the classification systems reviewed, only the Mental Health Care Clusters [34] used a cluster analysis to obtain profiles of patients. However, these profiles were developed on the basis of samples of patients with different psychiatric issues, such as depression or schizophrenia. As with age, dissimilarities across these groups will cancel out differences among individuals with dementia.

Implemented in daily practice in Switzerland, the Health of the Nation Outcome Scale for elderly people (HoNOS65+) [39, 40] assesses the global mental health, independently from the diagnosis. It allows the measurement and the follow-up of physical, personal and social issues associated to psychiatric disorders. Thus, the HoNOS65+ offers the opportunity to identify patient profiles based on multiple characteristics.

Consequently, the aim of our study was the development of profiles using cluster analysis of psychogeriatric patients hospitalized with dementia in Switzerland. The objectives were i) to find the optimal partition of patient profiles based on HoNOS65+ items as provided by cluster analysis; ii) to evaluate the validity, reliability, and clinical interpretation and meaningfulness of the final clustering solution; and iii) to describe the characteristics of each profile.

## Methods

### Design, sampling, settings, and data collection procedures

This study involved elderly patients with dementia who were hospitalized in one of three psychogeriatrics wards in French-speaking Switzerland between January 1, 2011, and June 30, 2014. Only the first hospitalization during the set period was taken into account.

Routinely collected data were used. These were under the responsibility of the head of the geriatric psychiatric service (AvG). Data were retrieved by a data manager who gave access to the data set after approval by the cantonal Human Research Ethics Committee (protocol no 231/14).

Subsequently, the main investigator (COB) met the canton’s centralized psychiatric hospital data manager to select subjects with a clinical diagnosis of dementia (according to the International Classification of Diseases, Tenth Revision) at admission to hospital. Clinical data was collected from the patients’ medical charts and the database containing all hospital stays in the cantonal psychogeriatrics wards during the study period; only initial stays were considered. Other medical data were retrieved to complete the clinical data that were not available in the computerized database. Reasons for hospital admission and the Mini-Mental State Examination (MMSE) [41] score at entry were collected in order to evaluate cognitive level. If the MMSE score was missing, the reason why that data was missing was recorded. We also noted the presence of comorbidities in order to calculate a comorbidity score using the Cumulative Illness Rating Scale for Geriatrics (CIRS-G) [42, 43]. Finally, the length of hospital stay in acute care was registered using the three cantonal medical insurance copayment categories found on patient charts: acute care, rehabilitation, and waitlisted for placement.

Of the 1104 patients meeting the inclusion criteria, 562 (51 %) had an incomplete HoNOS65+ assessment; this was due to a lack of time needed to complete the assessments at entry, making those assessments unusable for assessing the development of different profiles. For clinical purposes, HoNOS65+ assessments were made by at least two members of medical staff. Data about the remaining 542 (49 %) patients were used in our analysis. The two groups (with or without complete HoNOS65+ assessment) did not differ in age (81.34 vs. 81.49 years old; diff = −0.148 [95 % CI = −1.150–0.854]), CIRS-G score (19.43 vs. 18.70; diff = 0.727 [95 % CI = −0.069–1.522]), or MMSE score (17.42 vs. 16.64; diff = 0.778 [95 % CI = −0.304–1.860]).

With regards to this sample size, Dolnicar et al. [44] conducted simulations to estimate adequate sample sizes for market segmentation studies. According to the number of clusters, their separation index (amount of space between two clusters), and the number of noisy variables, the optimal sample size should range between 30*d* to 40*d*, where *d* is the number of variables included in the cluster analysis. A more conservative sample size requirement would be 70*d*. In the present study, nine variables and a sample size of 542 correspond to a sample size of 60*d* which was thus considered to be adequate.

### Development, validation, and selection of the best profile solution

Our cluster analysis was based on the French version of the HoNOS65+ [39, 45]. This scale is a diagnostic-independent assessment of mental health and social functioning. The 13 items were: (1) “behavioral disturbance”; (2) “non-accidental self-injury”; (3) “problem drinking or drug use”; (4) “cognitive problems”; (5) “problems related to physical illness or disability”; (6) “problems associated with hallucinations and/or delusions or false beliefs”; (7) “problems associated with depressive symptoms”; (8) “other mental and behavioral problems” (including phobias, anxiety and panic, obsessive-compulsive symptoms, mental strain and tension, dissociative and conversion problems, somatoform problems, eating disorder, sleep problems, sexual problems and other); (9) “problems with social or supportive relationships”; (10) “problems with activities of daily living”; (11) “overall problems with living conditions”; (12) “problems with work and leisure activities—quality of daytime environment”; and (13) “drug management”.

For each item, the observer chose the highest score applicable to the patient during the 2 weeks prior to the assessment. Each item was rated on a 5-point Likert scale as follows: 0 (no problem); 1 (a moderate problem requiring no action); 2 (a moderate but existing problem requiring monitoring and intervention for hospitalized patients), 3 (a moderately severe problem); 4 (a very severe problem) [46].

The scale showed good inter-rater reliability [47, 48], and associations between the individual HoNOS65+ items and other relevant established scales were generally adequate. For example: the HoNOS65+ behavioral disturbance item (item 1) was found to correlate with the Brief Agitation Rating Scale [39, 47]; the HoNOS65+ activity of daily living (ADL) item (item 10) was found to correlate with ADL, Instrumental ADL, and the Barthel rating scales for ADL [39, 40, 48]; the HoNOS65+ cognitive problems item (item 4) was found to correlate with MMSE scores [39, 47, 48]; the HoNOS65+ depression item (item 7) was found to correlate with the Geriatric Depression Scale [39, 47, 48]; and the HoNOS65+ physical illness item (item 5) was found to correlate with the CIRS-G [48].

To select relevant variables to be included in the cluster analysis, we examined each variable’s frequency, level of correlation, and whether it was properly representative of patients’ conditions. Based on this information, we used nine items of the HONOS65+ scale (items 1, 4–10, and 13). Item 2, “non-accidental self-injury”, and item 3, “problem drinking or drug use”, were not included because these problems were scarcely represented in aged patients with more advanced dementia. Item 11, “overall problems with living conditions”, and item 12, “problems with work and leisure activities—quality of daytime environment”, were not considered as they were more representative of patients living in a nursing home or at home alone.

**Fig. 1:**
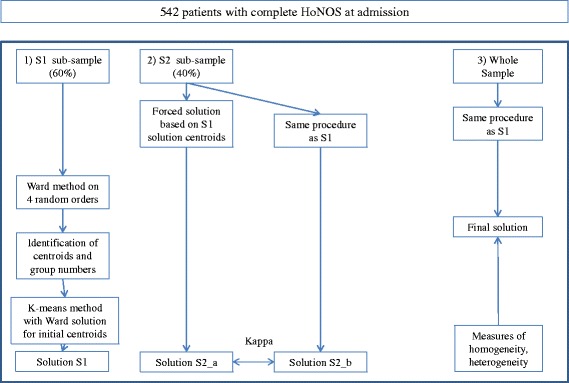
Illustration of the clustering procedure

In cluster analysis, hierarchical and non-hierarchical methods exist, each having advantages and disadvantages. The combination of both allows for the advantages of one approach to compensate for the weaknesses of the other. We applied the clustering process proposed by Hair [24] using a mixed approach combining hierarchical and non-hierarchical (k-means) cluster analysis. We also performed a *split-sample* cross-validation as proposed by Punj and Stewart [49] and calculated Kappa coefficients to test the reliability of the classification. This *split-sample* cross-validation procedure was performed simultaneously with the clustering process (Fig. 1).

For the cross-validation, the 542 subjects were randomly divided into two data sub-samples (S1 for the clustering development and S2 for the cross-validation, 60 and 40 % of patients each, respectively). On S1, we first applied Ward’s clustering hierarchical method using the Euclidian distance. We calculated solutions for three to seven clusters. Given the non-uniqueness of the solution in the agglomerative hierarchical cluster if ties exist [50], we performed the cluster analysis on four data permutations. Additionally, we compared the four solutions two-by-two by calculating percent agreements. If at least 70 % of observations fell in the same clusters at least in three of the four solutions, then the centroids of these observations were calculated in order to determine the initial cluster centroids. Second, the K-means method - a non-hierarchical procedure that clusters all observations using the initial clusters centroids solution from the hierarchical procedure - was used to provide more accurate cluster membership (solution S1).

Using sub-sample S2, clustering solutions were developed in two different ways to proceed to a cross-validation. First, the solution called S2_a was created by the classification of each observation of sub-sample S2 according to the nearest distance of the final centroids obtained from the k-means solution of sub-sample S1. Second, the S2_b solution was obtained independently repeating the mix-method approach (hierarchical and k-means) as described above for S1. These two alternative solutions (S2_a and S2_b) were then compared by calculating a Kappa agreement coefficient. The minimum threshold of 0.61 was used as the criterion for retaining a solution [51]. Third, the clustering process was repeated on the whole sample in order to obtain the final solution on which was computed the Calinski-Harabasz index with the aim to determine the optimal number of clusters (see below). This technique was applied on solutions from three to seven clusters (Fig. 1).

In addition to the Kappa coefficient used to evaluate reproducibility, we applied the Calinski-Harabasz index [52] to assess the ideal number of clusters. This statistical index is a weighted ratio of between- to within-cluster variance. If clusters are well-separated and compact, the between-cluster variance is large whereas the within-cluster variance is small. Thus, large values of the Calinski-Harabasz index are indicators of a better data partition. Compared to other criteria, this index is the most efficient [53] and is recommended by several authors [23, 24].

To assess the robustness of the final selected solution, we used the K-means method with 100 random initial seeds with the same number of clusters [54] as an alternative method. Among the 100 solutions, the one which minimized the sum of distances from observations to their centroid was retained. Memberships between the two solutions were examined using a Kappa coefficient.

In order to validate the final solution, differences in variables (between profiles) that were not used in the cluster analysis itself, but that are supposed to vary across profiles, were tested. For the present study, we selected the following variables: cognitive level measured by the MMSE [41], comorbidities evaluated using the CIRS-G [42, 43], length of stay in acute care, total length of hospital stay, and legal admission status. The Kruskall–Wallis, ANOVA and *χ*^2^ tests were used to compare the differences between clusters for continuous and categorical variables, respectively. All analyses were performed using in-house programming and the statistical software IBM SPSS Statistics, version 23 [55]. No special package was used.

## Results

### Description of the final profiles

Statistical properties of the solutions from three to seven clusters are illustrated in Table 1. The 4-cluster and the 5-cluster solutions showed the highest values of Calinski-Harabasz index and Kappa coefficient (267.41/0.83 vs 275.5/0.64 respectively). Considering that the Calinski-Harabasz index varied slightly between the two solutions unlike the Kappa coefficient which decreased substantially in the 5-cluster solution, the 4-cluster solution was finally selected. Furthermore, the 5-cluster solution did not provide more clinical information than the 4-cluster solution, and we easily observed in the data that the fifth profile in the 5-cluster solution was mainly the result of the division of one profile of the 4-cluster solution into two. These two profiles present similar patterns but slightly different levels of acuity.

**Table 1:** Statistical validation criteria of the solutions from three to seven clusters

	Calinski-Harabasz index	Kappa coefficient
3-cluster solution	308.26	0.41
4-cluster solution	267.41	0.83
5-cluster solution	275.50	0.64
6-cluster solution	221.99	0.57
7-cluster solution	200.54	0.55

Memberships between the two optimal 4-cluster solutions obtained by i) the mixed clustering approach with cross-validation, and by ii) the k-means method with 100 random initial seeds, were examined and showed a Kappa coefficient of 0.74, illustrating the robustness among methods for this solution.

**Fig. 2:**
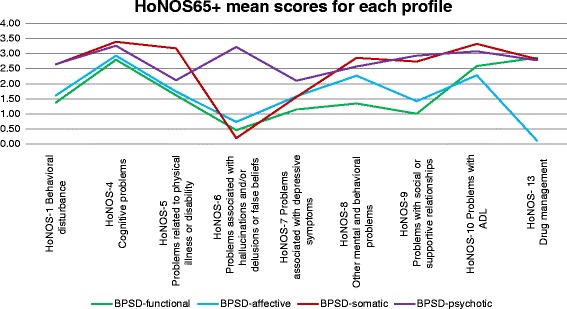
Illustration of the four clustered profiles. Profiles’ mean score on HoNOS65+ variables

As shown in Table 2 and illustrated in Fig. 2, each profile contained a reasonable proportion of patients (14–42 %). The nine items of the HoNOS65+ gave a maximum score of 36. Table 3 illustrated repartition details of HoNOS65+ item (8) “other mental and behavioral problems”. As shown in this table, anxiety was by far the most frequent problem reported by this item. Eating and sleeping disorders were also present but less prevalent whereas phobia, obsessive-compulsive symptoms, mental strain, dissociative and sexual problems were scarce in our study sample.

**Table 2:** Description of the four profiles

		BPSD-affective	BPSD-functional	BPSD-somatic	BPSD-psychotic
	n	233	95	137	77
HoNOS-1	mean (95%CI)	1.61 (1.45–1.77)	1.38 (1.14–1.62)	2.64 (2.46–2.82)	2.65 (2.38–2.92)
HoNOS-4	mean (95%CI)	2.94 (2.81–3.06)	2.80 (2.63–2.97)	3.39 (3.29–3.50)	3.26 (3.03–3.49)
HoNOS-5	mean (95%CI)	1.74 (1.58–1.90)	1.62 (1.37–1.87)	3.18 (3.03–3.32)	2.12 (1.80–2.44)
HoNOS-6	mean (95%CI)	0.74 (0.59–0.89)	0.46 (0.29–0.64)	0.20 (0.11–0.28)	3.22 (3.05–3.39)
HoNOS-7	mean (95%CI)	1.59 (1.43–1.75)	1.15 (0.92–1.38)	1.56 (1.35–1.77)	2.10 (1.82–2.38)
HoNOS-8	mean (95%CI)	2.27 (2.11–2.43)	1.35 (1.08–1.61)	2.86 (2.67–3.05)	2.57_b_ (2.29–2.86)
HoNOS-9	mean (95%CI)	1.42 (1.26–1.58)	1.01 (0.79–1.23)	2.74 (2.58–2.90)	2.94 (2.70–3.17)
HoNOS-10	mean (95%CI)	2.28 (2.14–2.42)	2.59 (2.42–2.76)	3.32 (3.21–3.43)	3.08 (2.83–3.33)
HoNOS- 13	mean (95%CI)	0.12 (0.07–0.16)	2.86 (2.73–2.99)	2.82 (2.67–2.97)	2.78 (2.52–3.04)
HoNOS- Total	mean (95%CI)	14.70 (14.13–15.28)	15.21 (14.55–15.88)	22.70 (22.21–23.20)	24.71 (23.79–25.63)

HoNOS-1: Behavioral disturbance

HoNOS-4: Cognitive problems

HoNOS-5: Problems related to physical illness or disability

HoNOS-6: Problems associated with hallucinations and/or delusions or false beliefs

HoNOS-7: problems associated with depressive symptoms

HoNOS-8:Other mental and behavioral problems

HoNOS-9: Problems with social or supportive relationships

HoNOS-10: Problems with ADL

HoNOS-13: Drug management

**Table 3:** Distribution of the HoNOS65+ item (8) “Other mental and behavioral problems” responses among the four profiles

HoNOS-8	Total *N* = 542	BPSD-affective *n* = 233	BPSD-functional *n* = 95	BPSD-somatic *n* = 137	BPSD-psychotic *n* = 77
Total of item 8 rated 1 or more, *n* (%)	443 (81.7)	195 (83.7)	56 (58.9)	124 (90.5)	68 (88.3)
A) Phobias	2 (0.5)	0 (0.0)	0 (0.0)	0 (0.0)	2 (2.8)
B) Anxiety and panic	294 (66.4)	133 (68.2)	43 (76.8)	74 (59.7)	44 (64.7)
C) Obsessive-compulsive symptoms	2 (0.5)	0 (0.0)	0 (0.0)	2 (1.6)	0 (0.0)
D) Mental strain and tension	7 (1.6)	6 (3.1)	0 (0.0)	0 (0.0)	1 (1.5)
E) Dissociative or conversion problems	1 (0.2)	1 (0.5)	0 (0.0)	0 (0.0)	0 (0.0)
F) Somatoform problems	1 (0.2)	1 (0.5)	0 (0.0)	0 (0.0)	0 (0.0)
G) Eating – over/under	43 (9.7)	18 (9.2)	2 (3.6)	12 (9.7)	11 (16.2)
H) Sleep – hypersomnia/insomnia	64 (14.4)	25 (12.8)	6 (10.7)	25 (20.2)	8 (11.8)
I) Sexual problems	6 (1.4)	2 (1.0)	1 (1.8)	3 (2.4)	0 (0.0)
J) Other	23 (5.2)	9 (4.6)	4 (7.1)	8 (6.5)	2 (2.9)

Based on ANOVA tests, total mean scores of HoNOS65+ differed between profiles (*F*(3538) = 218.86, *p* < 0.000). Post hoc comparisons using Tukey B revealed no differences between profiles 1 and 2 with relatively low total mean scores (respectively m = 14.70 and m = 15.22). Profiles 3 and 4 had high total mean scores (respectively m = 22.70 and m = 24.71) that were different from one another and from the mean scores of profiles 1 and 2. Based on clinical meaningfulness, we labeled the four profiles as *BPSD-affective*, *BPSD-functional*, *BPSD-somatic*, and *BPSD-psychotic*, respectively. The *BPSD-affective* and *BPSD-functional* profiles were different from the *BPSD-somatic* and *BPSD-psychotic* profiles in several HoNOS65+ items. People included in the *BPSD-affective* and *BPSD-functional* profiles showed fewer behavioral disorders, milder cognitive impairment, and fewer somatic problems than those in the *BPSD-somatic* and *BPSD-psychotic* profiles. The *BPSD-affective* profile was associated with mental health problems, mostly depression and anxiety, but not with psychotic symptoms. The *BPSD-functional* profile was mainly associated with a loss of independence in ADL and with the management of psychiatric drugs linked with difficulties in social relationships. Patients in the *BPSD-somatic* and the *BPSD-psychotic* profiles showed both a high level of cognitive impairment and dependence in ADL, more problems in social relationships and medication management, and presented other psychiatric disorders. However, people belonging to the *BPSD-somatic* profile had more somatic comorbidities than those belonging to the *BPSD-psychotic* profile, who experienced more psychotic symptoms.

Table 4 summarizes reasons for admission across profiles, and Table 5 summarizes other variables not included in the cluster analysis. The most frequent causes of admission to a psychogeriatric ward for patients with dementia were agitation, effective (or risk of) harm, and sheltering. Some causes were associated with specific profiles: delusional ideas and hallucinations were more frequent among patients in the *BPSD-psychotic* profile (*χ*^*2*^(3542) = 33.97, *p* < 0.000), and self-harm (whether a risk or real) was more frequent among patients characterized by the *BPSD-affective* profile (*χ*^*2*^(3542) = 9.36, *p* = 0.025). These differences were also judged to be clinically relevant.

**Table 4:** Causes of admission according to the four profiles

Reasons of admission	BPSD-affective *n* (%)	BPSD-functional *n* (%)	BPSD-somatic *n* (%)	BPSD-psychotic *n* (%)	*p*-value
Agitation	72	31	34	28	0.313
	(30.9 %)	(32.6 %)	(24.8 %)	(36.4 %)	
Harm (risk or effective)	67	23	45	29	0.228
	(28.8 %)	(24.4 %)	(32.8 %)	(37.7 %)	
Sheltering	38	23	22	13	0.339
	(16.3 %)	(24.2 %)	(16.1 %)	(16.9 %)	
Denial of care services	25	14	21	13	0.422
	(10.7 %)	(14.7 %)	(15.3 %)	(16.9 %)	
Delusional ideas and hallucinations	32	7	5	23	<0.001*
	(13.7 %)	(7.4 %)	(3.6 %)	(29.9 %)	
Care impossible at home	25	9	18	8	0.824
	(10.7 %)	(9.5 %)	(13.1 %)	(10.4 %)	
Self-harm (risk or effective)	33	10	9	3	0.025*
	(14.2 %)	(10.5 %)	(6.6 %)	(3.9 %)	

*p < 0.05 for *χ* ^2^ tests

**Table 5:** Variables not included in classification development

		BPSD-affective	BPSD-functional	BPSD-somatic	BPSD-psychotic	*p*-value
Gender		233	95	137	77	0.469
Men	*n* (%)	99 (42.48 %)	42 (44.21 %)	60 (43.79 %)	26 (33.76 %)	
Women	*n* (%)	134 (57.51 %)	53 (55.78 %)	77 (56.20 %)	51 (66.23 %)	
Age in years	*n*	233	95	137	77	0.074
	mean	80.98	80.84	82.94	80.21	
	(95 % CI)	(79.86–82.11)	(79.18–82.49)	(81.58–84.29)	(78.16–82.25)	
MMSE score	*n*	134	65	60	34	0.005*
	mean	18.49	17.06	15.03	18.12	
	(95 % CI)	(17.42–19.55)	(15.60–18.53)	(13.34–16.73)	(15.90–20.34)	
CIRS-G score	*n*	233	95	137	77	<0.001*
	mean	18.72	16.53	22.14	20.36	
	(95 % CI)	(17.92–19.51)	(15.31–17.74)	(21.05–23.23)	(18.84–21.88)	
Legal admission status	*n* total	233	95	137	77	0.004*
voluntary admission	*n* (%)	42 (18.02 %)	15 (15.78 %)	6 (4.37 %)	5 (6.49 %)	
non-voluntary admission	*n* (%)	188 (80.68 %)	79 (83.15 %)	129 (94.16 %)	72 (93.50 %)	
Other	*n* (%)	3 (1.3 %)	1 (1.1 %)	2 (1.5 %)	0 (0 %)	
Length of stay, acute Days	*n*	233	95	137	77	0.034*
	median	34	41	43	45	
	(IQR)	(37)	(40)	(32)	(40)	
Length of stay, total Days	*n*	233	95	137	77	0.070
	mean	55.47	72.18	58.61	62.16	
	(95 % CI)	(48.48–62.46)	(59.18–85.18)	(51.05–66.17)	(49.24–75.07)	

*p < 0.05 for ANOVA (means) and Kruskall-Wallis tests (medians)

The mean MMSE score also varied significantly across the profiles, with a lower score in the *BPSD-somatic* profile than in the *BPSD-affective* or *BPSD-psychotic* profiles (*F*(3289) = 4.435, *p* = 0.005). The MMSE scores of 46 % of patients who met the present study’s inclusion criteria are missing; this was mainly due to the difficulties of carrying out cognitive testing on acutely ill psychiatric patients or those with advanced dementia, or it was not considered clinically useful at hospital admission. Thus 46.5, 47.6, 29, 25 % of potential data was missing from the *BPSD-psychotic*, *BPSD-somatic*, *BPSD-affective*, and *BPSD-functional* profiles, respectively, because of the impossibility to carry out the MMSE evaluation.

The CIRS-G scores were also associated with particular profiles (*F*(3538) = 16.823, *p* < 0.000). Specifically, the *BPSD-somatic* profile had a higher comorbidity score than the *BPSD-affective* and *BPSD-psychotic* profiles, and the *BPSD-functional* profile presented the lowest comorbidity score.

Patients’ legal admission status (i.e. whether admission was voluntary or not) was also associated with the profiles (*χ*^*2*^(6542) = 19.38, *p* = 0.004). Non-voluntary hospital admissions made up significant proportions of all the profiles: 80 % for *BPSD-affective*; 83 % for *BPSD-functional*; 94 % for *BPSD-somatic*; and 93 % for *BPSD-psychotic*.

Finally, a Kruskal-Wallis test showed that the length of stay in acute care facilities varied across profiles (*p* = 0.034). Patients in the *BPSD-affective* profile had the shortest stays, with a median stay estimated at 34 days. The median stays for the other profiles varied between 41 and 45 days.

## Discussion

This study describes the development of four stable, valid, and reliable cluster profiles useful for distinguishing profiles of psychogeriatric patients hospitalized due to BPSD.

Our clustering solution allows to identify key issues for clinical management by combining agitation, psychological features (depression and psychotic symptoms), somatic disorders, and functional level. The identified profiles illustrate the complexity of the possible clinical situations presented by hospitalized psychogeriatric patients with cognitive impairment. Due to the specificity of these patients’ needs, the types of care and services they require should consider their complex multifactorial etiologies (e.g., [56]). It is important to note that the sample included in this cluster analysis is limited to patients hospitalized for BPSD as cluster analyses are sensitive to the characteristics of the studied population (34, 35). As our objective is to adapt care to the needs of the patients hospitalized in psychogeriatrics with BPSD, patients that differ from our population of interest should not be included in the analysis contrary to other classifications which have previously been developed based on samples including adult patients or various healthcare settings (acute, long-term stay or forensic).

The identified psychogeriatric profiles differed in terms of clinical levels of complexity and significantly explained variations in different clinical variables that have not been used for their development but these results can seem obvious : psychotic manifestations are a more frequent cause of admission in the *BPSD-psychotic* profile or contribute to a higher comorbidity score in the *BPSD-somatic* profile. These results are limited by the fact that the study was based on secondary data which constitutes its main limitation. Indeed, only routinely collected clinical data were accessible whereas a systematic evaluation including patients’ functional level, BPSD frequency and severity at admission would allow the identification of variables that can vary between profiles. Likewise, only few of the considered causes of admission were significantly different across the four profiles. However, the variables included in the development of the profiles took in account only patient-centered variables which would not be affected by external factors. The reasons that lead to hospitalization of the patient with dementia and BSPD certainly include not only clinical issues specific to the patient but also external factors, such as issues associated with living conditions or the caregiver’s health. As an example, a similar clinical situation can be managed at home for some BPSD patients but not for others, explaining why the reason of admission is not always associated with the profiles but more with external variables such as the availability of caregivers. Thus, it would be important for future research to examine the interactions between patient profiles, the living conditions and caregiver characteristics.

Two recent studies exploring our profiles shed light on the potential clinical perspectives. We explored the clinical course of these profile during hospitalization [57] and showed relative stability or improvement of patients: those who present *BPSD-functional* and *BPSD-affective* profiles remained in the same profile at discharge and those who present *BPSD-somatic* and *BPSD-psychotic* profiles showed clinical improvement during their hospitalization, transitioning to *BPSD-functional* or *BPSD-affective* profiles. Overall, the level of patients’ functional abilities remained the same during hospitalization as opposed to the decrease in BPSD. With regards to living arrangements after discharge, despite clinical improvement in most of the patients, the majority of them were institutionalized independently of the profile. Nevertheless, profiles were also associated with a specific living arrangement at discharge. Patients with a *BPSD-affective* profile had a higher probability of going back home and patients with a *BPSD-somatic* or *BPSD-psychotic* profile had a much higher probability of being hospitalized in acute wards or of dying compared to the *BPSD-functional* profile. This is evidence of a somatic decompensation. Patients with *BPSD-somatic* or *BPSD-psychotic* profiles displayed the most critical clinical health status of psychogeriatric inpatients compared to others with BPSD.

The aim of the second study was to describe the observations and nursing interventions that were recorded in the charts of the four profiles at admission. The study also aimed to identify the interventions considered most relevant for each profile through a structured exercise of consensus between experts. It helped to identify four types of interventions that are common to all profiles, and between two to five types of interventions specifically associated to each profile. The comparison of the interventions reported in the charts and those recommended by the experts offered potential perspective to improve nursing care in acute psychogeriatric wards [58].

This study has some limitations. As discussed above, due to the retrospective nature of this study, only routinely collected clinical data were accessible. With regard to external validity, it has not yet been possible to validate our findings on a different population sample. This raises the question of whether this profile solution could be applicable in other health care contexts. Indeed, a profile solution naturally depends to a great extent on the characteristics of the subjects classified and the organization of the healthcare system in place.

Thanks to the validity and reliability of our profiles in the present study, the future perspectives for research are multiple. Firstly, our solution can be used to develop a classification system. Indeed, based on the similarity (or distance) measure that has been used to develop the clustering solution, a new patient can be classified into a profile. Based on the values of the HoNOS65+ items of the new patient, the distance from each centroid of each group can be calculated, and the new patient can be assigned to the profile from which their distance to the centroid is minimal. This calculation algorithm could be easily developed and implemented in current electronic patient records. Moreover, it would be interesting to continue collecting clinical data associated with the profiles selected in order to more accurately determine their clinical course during hospitalization. Secondly, a systematic evaluation of patients’ functional level, the quality of their formal and informal networks, and their cognitive level at the different stages of hospitalization would be advantageous. Thirdly, with a more refined understanding of each profile, it might be possible to develop and test care-plan guidelines adapted to each one of them. Classifying patient according to clinical profiles could contribute to more easily identify the major issues in order to promptly plan tailored actions. According to the definition of the European Pathway Association, integrated care pathways are a “complex intervention to support decision making and organization of care expected for a group of well-defined patients during a determined period” [59]. Thus, integrated care pathways can be addressed to patient groups that can be defined by patient profiles sharing similar characteristics. Because of their attributes, integrated care pathways are renowned for being able to better focus care on patient needs [60–62], reduce the number of hospital readmissions and lengths of stay, whilst improving the quality, safety, and efficiency of care [61, 63–66]. This issue is important because integrated care pathways are mainly restricted to monitoring patients with an accurate medical diagnosis (such as diabetes or respiratory diseases) [61, 63, 67, 68]. There are currently no comprehensive care plans for managing patients with BPSD hospitalized in psychogeriatric wards.

## Conclusions

Aged patients with dementia are a heterogeneous population. The present study offers a four-profile solution which can serve to implement a new classification system for aged patients with BPSD according to their clinical characteristics when they are hospitalized in psychogeriatric units. These profiles have been validated statistically and clinically, and they are based on reliable clinical measurements systematically collected in daily practice. Because these profiles are associated with several clinical and psychosocial characteristics, they are likely to allow the development of patient-centered care plans.

## Abbreviations

ADL, activities of daily living; AN-SNAP, Australian National Subacute and Non-Acute Patient Classification; BPSD, behavioral and psychological symptoms of dementia; CIRS-G, cumulative Illness Rating Scale for Geriatrics; HoNOS65+, health of the nation outcome scales for elderly people; MMSE, Mini-Mental State Examination; NZ CAOS, New Zealand mental health Classification And Outcomes Study; SCIPP, System for Classification of In-Patient Psychiatry

## Additional file

Additional file 1: Review of classification systems developed for psychiatry. Summarizes several characteristics of existing classification systems that were developed for psychiatry. (XLSX 13 kb)
